# RNA Interference Targeting Testis-Specific Serine/Threonine Protein Kinase 1 (*TSSK1*) Gene Triggers Male Infertility in *Zeugodacus tau*

**DOI:** 10.3390/insects17050492

**Published:** 2026-05-12

**Authors:** Xinyao Huang, Wen Wen, Sihong Chen, Qiong Zhou, Wei Peng

**Affiliations:** Hunan Provincial Key Laboratory of Animal Intestinal Function and Regulation, State Key Laboratory of Developmental Biology of Freshwater Fish, Hunan International Joint Laboratory of Animal Intestinal Ecology and Health, Hunan Normal University, Changsha 410081, China

**Keywords:** *Zeugodacus tau*, *TSSK1*, RNAi, gSIT

## Abstract

*Zeugodacus tau*, a highly destructive agricultural quarantine pest causing severe economic losses to global fruit crops, urgently requires the development of male fertility-based control strategies. Here, we identified and characterized the testis-specific serine/threonine protein kinase 1 gene (*ZtTSSK1*) in *Z. tau*. The encoded protein of *ZtTSSK1* is highly conserved among dipteran species. Spatiotemporal expression analysis revealed predominant expression in adult males, with specific localization to the testes. In situ hybridization further localized *ZtTSSK1* transcripts to the transformation zone. Furthermore, functional characterization by RNA interference (RNAi) revealed that knockdown of *ZtTSSK1* resulted in a significant 62% reduction in sperm counts. While egg numbers laid by females mated to ds*ZtTSSK1*- versus ds*GFP*-injected males did not differ, hatching rates were significantly lower for eggs from ds*ZtTSSK1* matings. These findings establish *ZtTSSK1* as a key regulator of spermatogenesis and male fertility in *Z. tau*, providing a theoretical foundation and candidate target for genetic-based sterile insect technique (gSIT) development.

## Simple Summary

The genetic-based sterile insect technique (gSIT) represents an effective and environmentally friendly pest management strategy whose development depends on identifying key molecular targets governing fertility, particularly those involved in spermatogenesis. *Zeugodacus tau* (Walker), a highly polyphagous pest responsible for substantial economic losses in vegetable production, owes its rapid global expansion largely to high reproductive capacity. However, molecular targets suitable for gSIT implementation in this species remain largely unexplored. Here, we identified testis-specific serine/threonine protein kinase 1 (*ZtTSSK1*) as a gene significantly enriched during spermatid development in *Z. tau*. Using fluorescence in situ hybridization (FISH), we localized *ZtTSSK1* transcripts specifically to the transformation zone of the testis. Functional characterization via RNA interference (RNAi) bioassays demonstrated that suppression of *ZtTSSK1* reduced sperm counts and impaired male fertility. These findings identify *ZtTSSK1* as a promising molecular target for gSIT-based control of this economically important pest.

## 1. Introduction

The pumpkin fruit fly, *Zeugodacus tau* (Diptera: Tephritidae), is a highly polyphagous invasive pest that has established populations across tropical and subtropical Asia, sub-equatorial Africa, Australia, and the South Pacific region, causing substantial agricultural damage [1,2]. This pest attacks over 80 economically important crops, notably pumpkin, bitter gourd, angled luffa, and cucumber [3]. Females lay eggs beneath the epidermis of fruits and vegetables, and the larvae bore into the flesh, causing the fruit to rot. This damage not only directly reduces the yield of agricultural products but also causes secondary pathogen infections, further exacerbating the loss [4]. In China, the annual economic losses in the pumpkin industry attributable to *Z. tau* infestation are estimated at approximately CNY 23.2 billion [5,6]. Consequently, *Z. tau* is listed as a quarantine pest of international significance by China, the United States, Japan, Australia, Indonesia, New Zealand, and Pakistan [6,7,8]. Currently, tephritid fruit flies are primarily managed through chemical, biological, and physical methods, among which chemical insecticides are generally regarded as the most effective approach [9,10]. Organophosphates, neonicotinoids, and pyrethroids have shown effective control against *Z. tau* [11]. However, their overuse use has led to widespread ecological impacts, including environmental contamination, ecosystem disruption, persistent food residues, and the proliferation of pest resistance [12].

More recently, genetic-based control strategies such as the sterile insect technique (SIT) have shown promising potential for fruit fly population management [13,14]. It is a sustainable and eco-friendly method of pest management that complements other control strategies, providing long-term control of this economically important insect pest. SIT involves mass-rearing and sterilization of male insects, which are then released into the wild to mate with females. Mating with sterilized males leads to the laying of eggs by female insects that do not hatch, ultimately leading to a decline in the pest population [14,15,16]. The SIT has been successfully applied for fruit fly management in several countries, including China, Australia, and Hawaii [14,17]. In Hawaii, for example, SIT was implemented in the early 2000s to suppress populations of the oriental fruit fly, *Bactrocera dorsalis*, supporting the state’s agricultural sector. This initiative contributed to a marked reduction in pest prevalence [18]. Similarly, Australia has integrated SIT into its fruit fly pest management programs to help control the Mediterranean fruit fly, *Ceratitis capitata*, within horticultural production systems [19]. However, conventional SIT relies on radiation- or chemical-induced sterility, which can compromise male fitness and mating competitiveness. For example, irradiation damages testicular mitochondria and DNA, ultimately impairing sperm motility and insemination capacity in *Plutella xylostella* [20]. Consequently, identifying genes that regulate testis development is essential for optimizing SIT, and the development of precisely targeted genetic sterility techniques has emerged as a research priority.

Insect spermatogenesis is a tightly regulated process by which males produce mature sperm indispensable for egg fertilization [21]. This process encompasses spermatogonial proliferation and differentiation, meiosis of spermatocytes, and spermiogenesis [22,23]. During spermiogenesis, haploid round spermatids undergo morphological and functional transformation into elongated spermatids and fully mature spermatozoa. This process is characterized by acrosome formation, flagellum development, cytoplasmic reduction, chromatin condensation, and cellular elongation [24]. These extensive structural and functional changes are directed by precisely regulated gene transcription and protein translation, driven by complex molecular mechanisms within the testes, ultimately leading to the production of functionally mature sperm [25,26,27]. A growing body of research has sought to elucidate the genetic and molecular mechanisms governing spermatogenesis in insects, aiming to identify key genes as potential targets for SIT. Testis-specific *β-tubulin* genes, conserved across diverse insect taxa including *Bombyx mori*, *C. capitata*, and *Drosophila melanogaster*, are essential for spermatogenesis. In *D. melanogaster*, *β-tubulin* mutations disrupt microtubule function from meiosis onward, impairing motile sperm formation and causing defects throughout spermatogenesis [23,28,29,30,31,32,33,34,35,36,37]. In *Aedes aegypti*, RNAi knockdown of *ammonium transporter 1* (*Amt1*) reduces sperm count and egg viability [38], while CRISPR/Cas9 knockout of *leucine aminopeptidase 1* (*LAP1*) leads to mitochondrial defects, aberrant sperm autophagy, and impaired female fertility after mating [39]. Similarly, knockout of *TTLL3B* in *Bactrocera dorsalis* severely disrupts testis polyglycylation, causing mitochondrial malformation and paracrystalline accumulation, which markedly compromises male fertility [40].

Testis-specific serine/threonine protein kinases (*TSSKs*) are evolutionarily conserved in both mammals and insects, and are essential for sperm maturation and male fertility [41,42,43,44,45,46]. In *D. melanogaster*, TSSK mutations disrupted histone-to-protamine transition during spermiogenesis, impairing nuclear shaping, DNA condensation, and flagellar organization, which ultimately leads to severely reduced sperm motility and male infertility [47,48]. Furthermore, the *TSSKs* play a pivotal role in the spermatogenesis process of agricultural pests. RNAi-mediated knockdown of *TSSK1* in *B. dorsalis*, *Bactrocera tryoni*, and *Zeugodacus cucurbitae* similarly reduces sperm viability and male fertility, resulting in lower egg hatching rates [49,50,51]. In *Cydia pomonella*, disruption of *TSSKs* via RNAi and CRISPR/Cas9 also decreases sperm motility and count, and inhibits fertilized egg development [52].

RNA interference (RNAi) has emerged as a transformative tool for sustainable pest management. This technology relies on double-stranded RNA (dsRNA) to trigger sequence-specific gene silencing, allowing precise targeting of genes essential for insect survival or reproduction [53,54]. Owing to its design flexibility, target specificity, broad applicability, and favorable environmental compatibility, RNAi-based pesticides show significant potential as supplements or alternatives to conventional chemical insecticides. In recent years, the use of dsRNA for selective control of agricultural pests has gained considerable promise [55,56,57,58,59,60]. While microinjection is widely used for dsRNA delivery, it remains labor-intensive and requires specialized technical skills and equipment. To overcome these limitations, various oral delivery strategies have been successfully developed, including nanoparticle-mediated uptake and microbially expressed vector systems [61,62,63,64]. RNAi has demonstrated broad efficacy in controlling tephritid fruit flies and mosquitoes targeting vital genes. In *C. capitata*, simultaneous dsRNA targeting of *Vha68-1*, *dsRNase1*, and *dsRNase2* induced 79% mortality within seven days [65], while dsRNA against *V-ATPase* and *RPS13* reduced egg laying and hatching [66]. In other fruit flies, dsRNA treatments have enhanced mortality in *Anastrepha fraterculus* [67], improved RNAi efficacy by silencing gut nucleases in *B. tryoni* and *Z. cucurbitae* [68,69], and impaired reproduction in *Bactrocera oleae* and *B. dorsalis* [49,70,71]. Furthermore, RNAi targeting sex-determination pathways has effectively altered sex ratios and phenotypes. *Transformer-2* dsRNA treatment increased male proportion and induced intersex traits in *Zeugodacus scutellata* [72], and *transformer* and *transformer-2* DsiRNAs caused full masculinization of XX individuals in *C. capitata* [73]. Similarly, oral silencing of *doublesex* reduced fitness and lifespan in female *A. aegypti* and *Anopheles gambiae* [74,75]. Therefore, identifying and characterizing such genes essential for insect survival and reproduction is crucial for harnessing RNAi technology to reduce reliance on conventional chemical pesticides.

Given that the gene networks governing insect spermatogenesis are not yet fully understood, further identification and functional characterization of key regulators such as *TSSKs* will provide valuable targets for improving SIT-based pest management strategies. Previous studies have shown that *Tssk1* is expressed in mature spermatozoa and plays multiple roles in spermatogenesis in both mammals and insects [48,49,50,51,76]. While transcriptomic analysis of *Z. tau* testes previously identified 107 spermatogenesis-related orthologues [52], the functional role of *ZtTSSK1* in male fertility remains uncharacterized. Thus, we identify and characterize *ZtTSSK1* through sequence and expression analyses, and assess its role in male fertility using RNAi combined with fertility bioassays in *Z. tau*. Our findings not only demonstrate the role of *ZtTSSK1* in regulating spermatogenesis, but also identify it as a promising molecular target for genetic-based SIT (gSIT) strategies to control *Z. tau* populations.

## 2. Materials and Methods

### 2.1. Insect Rearing and Sample Collection

*Z. tau* pupae were collected in 2024 from Changsha, Hunan Province, China, and used to establish a laboratory colony maintained at 26 ± 1 °C under a 12 h light/12 h dark photoperiod in cages. Larvae were reared on pumpkin, and adults were fed an artificial diet containing 25% yeast powder and 75% sucrose. Embryos were obtained from females that were induced to oviposit on a piece of pumpkin for 10 min, with the first egg batch being discarded. Each development stage sample of *Z. tau* contained three biological replicates, and each replicate contained 100 embryos, 20 larvae, 20 pupae, and 5 adults. The head, thorax, midgut, Malpighian tubules, fat body, and ovaries (from females) or testes (from males) were dissected and collected. Samples were transferred to nuclease-free centrifuge tubes and immediately frozen in liquid nitrogen.

### 2.2. Sequence Alignment and Phylogenetic Analysis

The deduced amino acid sequences of ZtTSSK1 and homologous proteins from other insect species were obtained through NCBI BLASTP searches. Multiple sequence alignment was performed using DNAMAN 9.0 software with the ClustalW algorithm, and pairwise sequence identity values were calculated. Phylogenetic trees were constructed from the deduced ZtTSSK1 amino acid sequence obtained in this study, together with those from indicated species, using the MEGA7 program with the neighbor-joining algorithm. Branch support was assessed by bootstrap analysis with 1000 replicates.

### 2.3. Fluorescence In Situ Hybridization

A FAM-labeled *ZtTSSK1* probe with the sequence 5′-CAAATTGGCTGATTTCGGCTTTGCACGCTATTGCTGCGACGATAG-3′ was synthesized in vitro by Sangon Biotech (Shanghai, China). For fluorescence in situ hybridization (FISH), testes were dissected from a 5-day-old adult *Z. tau* in phosphate-buffered saline (PBS) and fixed overnight at 4 °C in 4% paraformaldehyde. Fixed testes were subsequently washed three times with PBST (2% Triton X-100 in PBS) (Solarbio, Beijing, China) for 5 min each, treated with 0.25% hydrochloric acid for 30 min, washed again three times with PBST (5 min each), and incubated with proteinase K for 20 min at room temperature. Following three additional PBST washes (5 min each), the testes were incubated in pre-hybridization solution for 60 min at 68 °C. The testes were then incubated with the prepared probes (diluted 500×) in the dark at 68 °C for 24 h. After three final washes with PBST (15 min each), testes were stained with DAPI for 10 min and mounted on slides. Fluorescence images were acquired using a Zeiss AxioImager M2 microscope (Carl Zeiss MicroImaging, Oberkochen, Germany).

### 2.4. Synthesis and Injection of Double-Strand RNA (dsRNA)

A *ZtTSSK1* fragment was amplified by PCR from male adult cDNA, and a control *GFP* fragment was generated using primers derived from the *GFP*-containing pNCS-mNeonGreen vector. Double-stranded RNA (dsRNA) corresponding to *ZtTSSK1* and *GFP* fragments was synthesized with the T7 RiboMAX^TM^ Express RNAi System (Promega, Madison, WI, USA). The primer sequences are listed in Table S1. The dsRNA was purified using 3M Sodium Acetate (pH 5.2) (Thermo Fisher Scientific, Waltham, MA, USA) and isopropanol, dissolved in injection buffer (5 mM KCl, 0.1 mM sodium phosphate, pH 6.8), and its quality was verified by 1.2% agarose gel electrophoresis. The final concentration of dsRNA was adjusted to 2 μg/μL. Microinjection was performed using a Nanoject III device (Drummond Scientific Company, Broomall, PA, USA). A total of 60 newly eclosed virgin males were each injected with 1 μg of ds*ZtTSSK1* or ds*GFP* (500 μL) into the ventral thorax (three biological replicates). Five flies per sample were collected for RNA isolation and analyses of RNAi efficiency at 24, 48, and 72 h post-injection.

### 2.5. Quantitative Real-Time PCR

Candidate-gene transcript levels were quantified by quantitative real-time PCR (qRT-PCR). Total RNA was isolated using RNAiso Plus reagent (TaKaRa, Dalian, China) per replicate, and 200 ng of each sample was reverse-transcribed using PrimeScript^TM^ RT Master Mix (TaKaRa, Dalian, China). The resulting cDNA was amplified by qRT-PCR with gene-specific primers (Table S1) using 480 SYBR^®^ Green (Roche, Basel, Switzerland) on a Bio-Rad real-time thermal cycler (Hercules, CA, USA) under the following conditions: 95 °C for 10 min, followed by 40 cycles of 95 °C for 15 s, 60 °C for 30 s, and 72 °C for 30 s. Each reaction was performed in triplicate (three biological and three technical replicates). Relative expression was calculated using the 2^−ΔΔCt^ method [77]. Melting-curve analysis confirmed single-product amplification for each transcript, with *rpl32* serving as the internal reference gene.

### 2.6. Sperm Motility and Quantification Test

A single biological replicate consisted of testes pairs from two individuals, with three replicates prepared for both ds*ZtTSSK1*-injected and ds*GFP*-injected 9-day-old *Z. tau* males. Testes were dissected and pierced with forceps, and 2 μL of sperm-containing fluid was collected. This liquid was diluted with 300 μL of buffer, and sperm activity was measured using the LIVE/DEAD^TM^ Sperm Viability Kit (Thermo Fisher Scientific, Waltham, MA, USA) as described previously [35,37]. Briefly, 5 μL of diluted SYBR14 solution was added and incubated for 10 min at 36 °C, followed by the addition of 5 μL of propidium iodide and incubation for 10 min at room temperature. Green (live) and red (dead) sperm were then counted using a Zeiss Image M2 fluorescence microscope (Carl Zeiss MicroImaging, Oberkochen, Germany). Additionally, the testes and sperm were stained with DAPI and mounted on slides. Total sperm numbers were counted as described previously [50].

### 2.7. Reproduction Bioassays

Thirty ds*ZtTSSK1*- or ds*GFP*-injected *Z. tau* males were maintained separately on a standard artificial diet for 9 days, then transferred to three 30 cm × 30 cm × 40 cm cages with ten age-matched virgin females to allow copulation and oviposition. Upon completion of copulation, males were removed, while females were retained for subsequent fecundity assays with three biological replicates. Eggs were collected using a trap containing a fresh pumpkin slice. The slice with deposited eggs was transferred to a Petri dish lined with moist filter paper to maintain humidity. Egg-hatching rates were assessed 48 h after collection.

### 2.8. Statistical Analysis

All qRT-PCR experiments were performed with at least three independent biological replicates. Data were analyzed using GraphPad Prism 8.0 (GraphPad Software, San Diego, CA, USA) or Microsoft Excel (Microsoft, Redmond, WA, USA) and are presented as mean ± SEM. Group comparisons were made by one-way ANOVA followed by Bonferroni’s post-hoc test for multiple comparisons, or by Student’s *t*-test where appropriate. Statistical significance was set at *p* < 0.05.

## 3. Results

### 3.1. Sequence Analyses and Phylogenetic Tree Construction of ZtTSSK1 Gene

**Figure 1:**
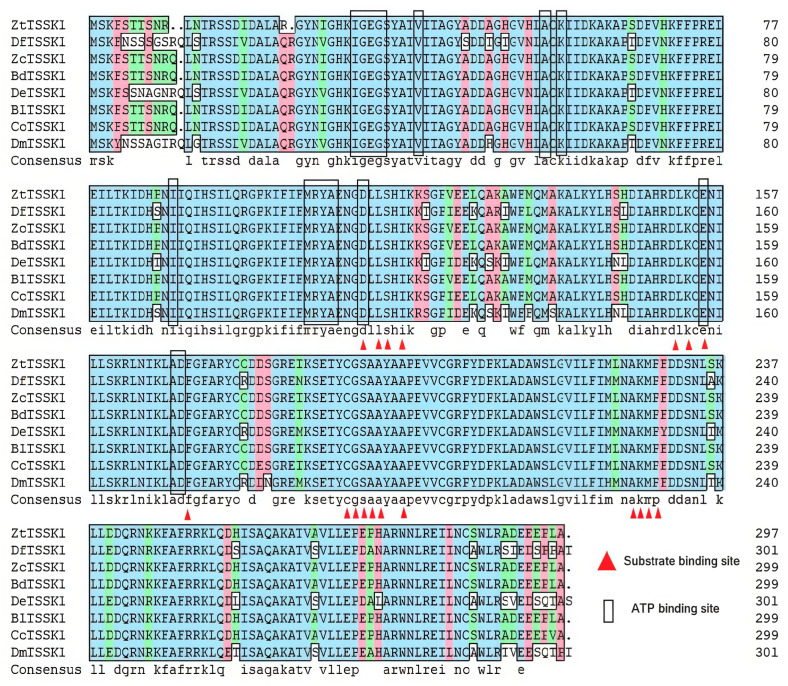
Multiple sequence alignment of ZtTSSK1 amino acids in *Z. tau*. Multiple sequence alignment of TSSK1 amino acids from *Bactrocera dorsalis* (XP_011211029.1), *Bactrocera latifrons* (XP_018803871.1), *Ceratitis capitata* (XP_004536887.1), *Drosophila elegans* (XP_017113760.1), *Drosophila ficusphila* (XP_017039897.1), *Drosophila melanogaster* (NP_650732.1), *Zeugodacus cucurbitae* (XP_011192630.1), *Zeugodacus tau* (CM062649.1). Identical amino acids are shaded in blue (the homology is 100%) while similar amino acids are shaded in red (the homology is greater than or equal to 75%) and green (the homology is greater than or equal to 50%). The alignment method was ClustalW and the multiple sequence alignment was constructed by using DNAMAN software. ATP binding sites and substrate binding sites are respectively represented with black lines and red triangles.

**Figure 2:**
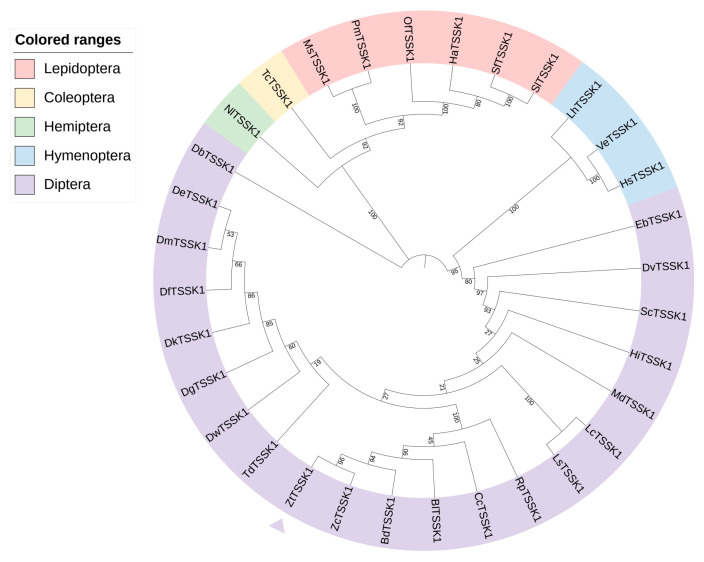
Phylogenetic analysis of *TSSK1* in *Z. tau.* Neighbor-joining trees of insect TSSK1 amino acid sequences. The numbers next to the branches represent bootstrap support value from 1000 replicates. The scale represents the mean character distance. The topology was rooted with the TSSK1 protein from *Bactrocera dorsalis* (XP_011211029.1), *Bactrocera latifrons* (XP_018803871.1), *Ceratitis capitata* (XP_004536887.1), *Drosophila busckii* (XP_017847448.1), *Drosophila elegans* (XP_017113760.1), *Drosophila ficusphila* (XP_017039897.1), *Drosophila guanche* (XP_034137421.1), *Drosophila kikkawai* (XP_017035418.1), *Drosophila melanogaster* (NP_650732.1), *Drosophila virilis* (XP_002056229.1), *Drosophila willistoni* (XP_002073536.1), *Episyrphus balteatus* (XP_055848576.1), *Haematobia irritans* (XP_075147315.1), *Harpegnathos saltator* (XP_011145333.3), *Helicoverpa armigera* (XP_021183931.1), *Leptopilina heterotoma* (XP_043461997.1), *Lucilia cuprina* (XP_023305836.2), *Lucilia sericata* (XP_037806454.1), *Manduca sexta* (XP_037297243.1), *Musca domestica* (XP_005186105.1), *Nilaparvata lugens* (XP_022193368.2), *Ostrinia furnacalis* (XP_028163804.1), *Papilio machaon* (XP_014361.1), *Rhagoletis pomonella* (XP_036324222.1), *Spodoptera frugiperda* (XP_035431594.1), *Spodoptera litura* (XP_022828857.1), *Stomoxys calcitrans* (XP_013116648.1), *Teleopsis dalmanni* (XP_037952881.1), *Tribolium castaneum* (XP_008192371.1), *Vollenhovia emeryi* (XP_011882868.1), *Zeugodacus cucurbitae* (XP_011192630.1), *Zeugodacus tau* (CM062649.1). The MEGA7 program was used for the construction of phylogenetic trees with the neighbor-joining algorithm. The triangle indicates the TSSK1 protein from *Z. tau*.

The sequence of the *TSSK1* gene was obtained from the *Z. tau* genome database (https://www.ncbi.nlm.nih.gov/datasets/genome/?taxon=137263) (accessed on 10 May 2024) and *ZtTSSK1* comprises an open reading frame encoding 299 amino acids. ClustalW multiple sequence alignment revealed that *ZtTSSK1* shares high homology with *TSSK1* orthologues from other insects, with highly conserved residues in the serine/threonine kinase catalytic (S-TKc) domain and both adenosine triphosphate (ATP)- and substrate-binding sites, indicative of ATP-binding activity (Figure 1). The alignment results indicate that *TSSK1* of *Z. tau* shares 84–100% identity with other insects (Figure 1). Phylogenetic analysis placed *ZtTSSK1* within a dipteran clade comprising *B. dorsalis*, *Bactrocera latifrons*, *Ceratitis capitata*, *Rhagoletis pomonella*, and *Z. cucurbitae*, followed by separate branches for Lepidoptera, Hymenoptera, Hemiptera, and Coleoptera. *ZtTSSK1* was most closely related to *ZcTSSK1* from *Z. cucurbitae* (Figure 2).

### 3.2. Expression Patterns of ZtTSSK1 Gene

**Figure 3:**
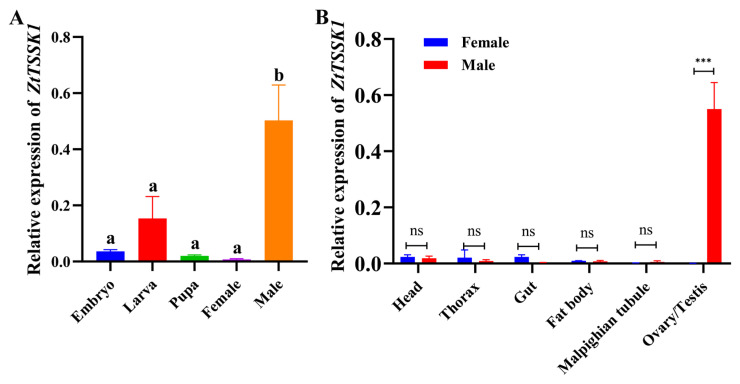
Temporal and spatial expression pattern analysis of *ZtTSSK1* in *Z. tau*. (**A**) The relative expression of *ZtTSSK1* from different developmental stages. (**B**) The relative expression of *ZtTSSK1* from different tissues. Error bars indicate SEM of three independent biological replicates. The different letters above the error bars indicate statistically significant differences (*p* < 0.05) based on ANOVA with Tukey’ s HSD post-hoc test at different development stages. Asterisks (***) indicate statistically significant differences (*p* < 0.001) between male and female tissues based on Student’s *t*-test, and ns indicates no statistical difference.

To verify the role of *ZtTSSK1* in the regulation of spermatogenesis in *Z. tau*, expression profiles of *ZtTSSK1* were measured using qRT-PCR at different developmental stages and in different tissues. The results showed that *ZtTSSK1* mRNA was expressed in all stages including embryos, larvae, pupae, and adults, with levels significantly higher in adult males (Figure 3A). The expression profile of *ZtTSSK1* in different tissues showed that *ZtTSSK1* mRNA was exclusively expressed in the adult testes (*p*-value, 0.0005) (Figure 3B), indicating that *ZtTSSK1* likely plays a pivotal role in the spermatogenesis of *Z. tau*.

### 3.3. FISH Analyses of the Localization of ZtTSSK1 Within the Testis

**Figure 4:**
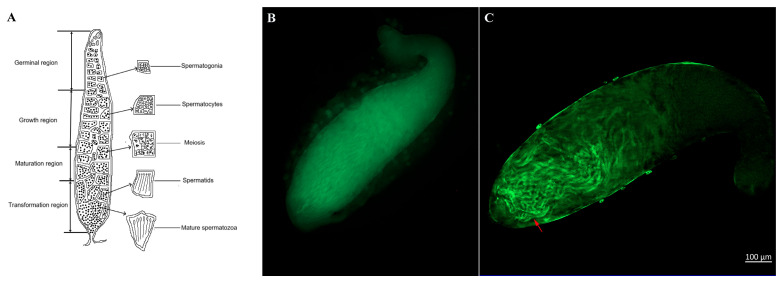
*ZtTSSK1* localization in the testis of *Z. tau*. (**A**) The schematic diagram of subregion in testis of *Z. tau*. (**B**) The negative control FISH signal in the testis of *Z. tau*. (**C**) The fluorescence signal for *ZtTSSK1* in the testis of *Z. tau* (red arrow). The scale bar is 100 μm and indicated in the bottom right corner.

The spatial expression of *ZtTSSK1* in adult *Z. tau* testes was examined by FISH. The results showed that *ZtTSSK1* signals were detected in the transformation region, predominantly concentrated in mature sperm bundles and spermatids (Figure 4B,C and Figure S1). This region, which connects to the vas deferens, serves as the site where sperm undergo morphological transformation and maturation (Figure 4A).

### 3.4. Functional Analyses of the Roles of ZtTSSK1 in Male Fertility

**Figure 5:**
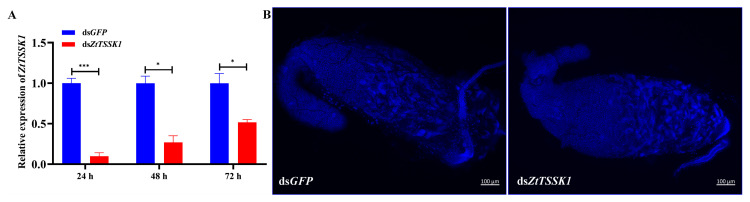
Evaluation of ds*ZtTSSK1* RNAi efficiency in *Z. tau*. (**A**) The expression level of *ZtTSSK1* after RNAi knockdown 24 h, 48 h, and 72 h. *ZtTSSK1* was significantly reduced by 90%, 73%, and 48% at 24 h, 48 h, and 72 h, respectively. (**B**) Morphology of testes after injection of ds*GFP* or ds*ZtTSSK1*. The scale bar is 100 µm as shown in the figure. Data are presented as mean ± SEM. Difference between two groups was tested using an unpaired *t*-test, with * indicating *p* < 0.05, *** for *p* < 0.001.

**Figure 6:**
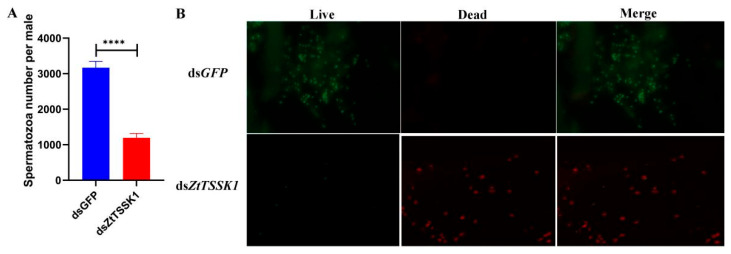
Reduced number of spermatozoa in *ZtTSSK1* knockdown males. (**A**) *Z. tau* spermatozoa numbers following RNAi. Data are presented as mean ± SEM. Difference between two groups was tested using an unpaired *t*-test, with **** for *p* < 0.0001. (**B**) Represents the percentage of live spermatozoa in males injected with ds*GFP* and ds*ZtTSSK1*. Red indicates dead sperms, green indicates live sperms.

**Figure 7:**
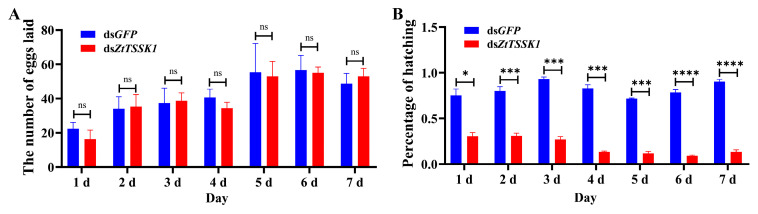
Effects of RNAi-mediated suppression of *ZtTSSK1* on male fertility. (**A**) The impact of *ZtTSSK1* knockdown on oviposition in *Z. tau*. (**B**) The impact of *ZtTSSK1* knockdown on egg hatching rates in *Z. tau*. Error bars indicate SEM of three independent biological replicates. Difference between two groups was tested using an unpaired *t*-test, with * indicating *p* < 0.05, *** indicating *p* < 0.001, **** indicating *p* < 0.0001, and ns indicating no statistical difference.

The expression levels of *ZtTSSK1* mRNA in a male *Z. tau* injected with ds*ZtTSSK1* were examined at 24 h, 48 h, and 72 h post-injection using qRT-PCR (Figure 4). The relative expression of *ZtTSSK1* was significantly reduced by 90% (*p*-value, 0.0002), 73% (*p*-value, 0.0102), and 48% (*p*-value, 0.0172) at 24 h, 48 h, and 72 h compared to ds*GFP*-treated controls, respectively (Figure 5A). Besides, the cycle threshold (Ct) values of *rpl32* gene had no significant difference between ds*ZtTSSK1*- and ds*GFP*- injected males at 24 h (*p*-value, 0.7356) (Figure S2). Collectively, these results validate the effectiveness of RNAi-mediated knockdown of *ZtTSSK1* in *Z. tau*. To assess how *ZtTSSK1* silencing affects reproductive fitness in *Z. tau*, we performed testis morphological observation and sperm viability assays at 9 days post-injection. The results showed that dsRNA injection had no impact on testis morphology in either ds*ZtTSSK1*- or ds*GFP*-injected males (Figure 5B). This may be attributed to the fact that the testes of male *Z. tau* complete their development shortly after adult eclosion [52]. However, ds*ZtTSSK1*-treated males exhibited a significant 62% reduction in sperm counts compared to the control group (*p*-value, <0.0001) (Figure 6A). The number of sperms in ds*ZtTSSK1*- and ds*GFP*-treated males is 3168 and 1194, respectively (Figure 6A). Interestingly, eighty-nine percent of spermatozoa in ds*ZtTSSK1*-injected males were dead, whereas all spermatozoa in ds*GFP*-injected males were viable (Figure 6B). These findings indicate that *ZtTSSK1* disruption impairs male fertility through compromised sperm viability. To investigate the effects of *ZtTSSK1* silencing on the reproductive capacity of *Z. tau* males, we further monitored daily egg production and performed hatching assays. The results showed that egg numbers did not differ between females mated to ds*ZtTSSK1*- versus ds*GFP*-injected males (Figure 7A). However, hatch rates were significantly lower for eggs from ds*ZtTSSK1* matings (*p*-value, 0.0177, 0.0009, 0.0004, 0.0009, 0.0002, <0.0001, <0.0001) (Figure 7B), and ds*ZtTSSK1*-injected males showed 98% reduction in viable progeny relative to ds*GFP*-injected males (Figure S3).

## 4. Discussion

In this study, we identified and characterized the testis-specific serine/threonine protein kinase gene *ZtTSSK1* in *Z. tau* through sequence analysis, expression profiling, and functional assays, revealing its crucial role in male reproduction and identifying a promising candidate target for gSIT control of this pest.

*ZtTSSK1* contains the S-TKc domain and ATP- and substrate-binding sites, motifs highly conserved across insects including *Anopheles stephensi*, *B. mori*, *C. pomonella*, *D. melanogaster*, *P. xylostella*, and *Z. cucurbitae* [47,50,51,52,76,78,79,80]. The TSSK family belongs to the adenosine monophosphate-activated protein kinase (AMPK) family, with the S-TKc domain mediating serine/threonine protein kinase activity. This domain establishes the correct spatial conformation and catalyzes the transfer of the γ-phosphate group from ATP to target proteins [81]. Sequence alignment revealed that lysine and aspartate residues required for ATP binding and catalytic activity are conserved in *ZtTSSK1* and other *TSSK1* orthologues. Phylogenetic analysis reveals that *ZtTSSK1* clusters within a conserved dipteran clade, distinct from those of Lepidoptera, Hymenoptera, Hemiptera, and Coleoptera, and shows a close relationship to the corresponding protein from *Z. cucurbitae*.

Stage- and tissue-specific profiling revealed that *ZtTSSK1* is highly expressed in adult male testes of *Z. tau*, consistent with testis-restricted *TSSK1* expression reported in *B. dorsalis*, *B. tryoni*, *C. pomonella*, and *Z. cucurbitae* [48,49,50,51]. This pattern indicates a conserved role for *TSSK1* in spermatogenesis across diverse insects. FISH further localized *ZtTSSK1* predominantly to the transformation zone, where spermatids differentiate into flagellated spermatozoa, a distribution matching that of *ZcTSSK1* in *Z. cucurbitae* [50]. These congruent expression and localization patterns strongly suggest that *ZtTSSK1* and *ZcTSSK1* serve analogous functions as key mediators of spermatogenesis. Similar findings have been reported for the testis-specific gene lnc94638, which is likewise enriched in the transformation zone, and its knockdown impairs male fertility in *Z. cucurbitae* [82]. In *B. mori*, *TSSK3* is testis-specific and localized to sperm flagella, with pronounced accumulation in the sperm tail cyst [67], whereas in *D. melanogaster*, *TSSK2* is testis-restricted and associated with individualization complexes during spermiogenesis [47]. Collectively, these data indicate that *ZtTSSK1* functions as a post-meiotic regulator influencing spermatogenic progression in *Z. tau* males.

RNA interference (RNAi) was employed to knock down *ZtTSSK1* in male *Z. tau*, and dsRNA injection significantly reduced *ZtTSSK1* transcript levels. This knockdown markedly reduced sperm numbers and the egg-hatching rates of females mated with *ZtTSSK1*-silenced males. Given the critical role of *TSSKs* in regulating chromatin remodeling during the post-meiotic stage in males [47,76], disruption of *ZtTSSK1* expression likely results in sperm malformation and reduces the number of mature spermatozoa. Similar fertility defects have been observed following *TSSK* gene knockdown in other insect species, including *A. stephensi*, *B. dorsalis*, *B. tryoni*, *C. pomonella*, *D. melanogaster*, *P. xylostella*, and *Z. cucurbitae* [47,48,49,76,79,80]. RNAi-mediated *TSSK1* knockdown in *B. dorsalis*, *B. tryoni*, and *Z. cucurbitae*, and *TSSK3* knockdown in *A. stephensi* via dsRNA feeding, reduced sperm motility and induced male sterility [48,49,50,80]. In *C. pomonella*, *TSSK1* knockdown and knockout using dsRNA injection and CRISPR/Cas9 editing, respectively, disrupted spermiogenesis, decreased sperm motility, and hindered egg development [51]. In *D. melanogaster*, mutations in *TSSK* genes disrupted the histone-to-protamine transition during spermiogenesis, severely impaired sperm motility, and resulted in male sterility [46,47]. CRISPR/Cas9-mediated *TSSK3* deletion in *P. xylostella* caused testis developmental defects, sperm death, and male sterility. Furthermore, deficiency of *PxTSSK3* led to significant enrichment of potential phosphorylation substrates in pathways associated with spermatogenesis, microtubule cytoskeleton organization, and histone modification [79]. Additionally, *TSSK3* knockdown significantly reduced histone H3 phosphorylation in *B. mori* [78]. Together, these results emphasize the conserved function of *TSSKs* in regulating male fertility among diverse insect species, while also indicating that the specific molecular mechanisms by which individual *TSSKs* exert their effects may differ.

Conventional SIT relies on irradiation or chemical sterilants that reduce pest populations but often compromise male mating competitiveness and fitness [14,20]. In contrast, gSIT targeting key male reproductive genes achieves sterility without affecting male competitiveness, thereby markedly improving control efficiency. *ZtTSSK1*, which is expressed exclusively in the testes and is essential for male fertility, fulfills the core criteria of a gSIT target: its knockdown blocks reproduction without noticeable effects on growth or development. SIT programs releasing males rendered sterile via *ZtTSSK1* disruption can interrupt the reproductive cycle of wild populations, offering a novel approach for area-wide control. RNAi-mediated knockdown of *ZtTSSK1* in our study only significantly reduced egg hatchability without achieving complete male sterility, an outcome comparable to single *TSSK* gene knockdown in *B. dorsalis*, *B. tryoni*, *C. pomonella*, and *Z. cucurbitae* [48,49,50,51]. Sterility rates in *TSSk1* knockdown males reached 59% in *B. dorsalis* and 78% in *B. tryoni* [48,49]. In *C. pomonella*, knockdown of *TSSK1*, *TSSK1a*, *TSSK2*, *TSSK2a*, and *TSSK4* resulted in sterility rates of 91%, 84%, 100%, 100%, and 100%, respectively [51]. Partial sterility was also observed following *TSSK1* and *TSSK3* knockdown in *Z. cucurbitae* [50]. Functional redundancy among *TSSK* paralogues, or the need for simultaneous targeting of additional reproductive regulators, may account for this partial effect. Previous studies have demonstrated that combining gene silencing with low-dose irradiation can further suppress fertility without impairing male mating success, thereby enhancing SIT efficacy [78]. Future research should therefore investigate synergistic knockdown of *ZtTSSK1* together with other *TSSK* members or spermatogenesis-related genes, or incorporate low-dose radiation to optimize sterile-male production protocols. Furthermore, the precise molecular mechanisms by which *ZtTSSK1* governs spermatogenesis, such as its downstream phosphorylation substrates and interactions with other signaling pathways, remain to be elucidated. Conventional irradiation-based SIT often produces sterile males with reduced mating competitiveness. In contrast, strategies aimed at generating sterile yet highly competitive males have become a promising alternative for population control [39,51]. Ideally, sterility-inducing genetic modifications could spread naturally through pest populations without requiring continuous mass releases of sterile insects [83]. Therefore, mating with *TSSKs* mutant males induced by RNAi or CRISPR/Cas9 represents an effective gSIT strategy, as it allows genetic modifications to disseminate within pest populations via a reduced number of viable offspring [53,54].

## 5. Conclusions

The present study demonstrates that *ZtTSSK1* plays a critical role in the male fertility of *Z. tau*. RNAi-mediated knockdown significantly reduced *ZtTSSK1* transcript levels, severely compromising reproductive capacity through diminished sperm production. The resulting decline in sperm quantity and quality ultimately led to male sterility. These findings not only expand our understanding of the molecular framework underlying insect reproductive physiology, but also establish a basis for developing gSIT through targeted genetic modification strategies.

## Data Availability

The original contributions presented in this study are included in the article/Supplementary Materials. Further inquiries can be directed to the corresponding authors.

## Supplementary Materials

The following supporting information can be downloaded at: https://www.mdpi.com/article/10.3390/insects17050492/s1, Figure S1: Quantification of *ZtTSSK1* signal intensity in the testis of *Z. tau*. Figure S2: The Ct values of *RpL32* in males injected with ds*GFP* and ds*ZtTSSK1*. Figure S3: Percentage of males injected with ds*GFP* and ds*ZtTSSK1* exhibiting sterility. Table S1: Primers used in our study.

## References

[1] Jaleel W., Yin J., Wang D., He Y., Lu L., Shi H. (2018). Using two-sex life tables to determine fitness parameters of four *Bactrocera* species (Diptera: Tephritidae) reared on a semi-artificial diet. Bull. Entomol. Res..

[2] Li X., Yang H., Hu K., Wang J. (2020). Temporal dynamics of *Bactrocera* (Zeugodacus) *tau* (Diptera: Tephritidae) adults in north Jiangxi, a subtropical area of China revealed by eight years of trapping with cuelure. J. Asia-Pac. Entomol..

[3] Liu P., Zheng W., Qiao J., Li Z., Deng Z., Yuan Y., Zhang H. (2022). Early embryonic transcriptomes of *Zeugodacus tau* provide insight into sex determination and differentiation genes. Insect Sci..

[4] Huang Y., Gu X., Peng X., Tao M., Chen G., Zhang X. (2020). Effect of short-term high-temperatures on the growth, development and reproduction in the fruit fly, *Bactrocera tau* (Diptera: Tephritidae). Sci. Rep..

[5] Su H., Zhao J., Yu H., Jaffar S., Hao Z., Liang G., Zeng L., Lu Y. (2025). Oviposition competition between *Zeugodacus cucurbitae* and *Bactrocera dorsalis* adults in five hosts. Insects.

[6] Fang Y., Li Z., Qin M., Wu Z., Zhao S., Wu L., Zhao Z., Chen K., Qin Y.J., Wang C. (2015). The potential economic impact of the pumpkin industry caused by *Bactrocera tau* (Walker). Plant Quar..

[7] Ohno S., Tamura Y., Haraguchi D., Kohama T. (2008). First detection of the pest fruit fly, *Bactrocera tau* (Diptera: Tephritidae), in the field in Japan: Evidence of multiple invasions of Ishigaki Island and failure of colonization. Appl. Entomol. Zool..

[8] Shi W., Kerdelhué C. (2014). Genetic structure and colonization history of the fruit fly *Bactrocera tau* (Diptera: Tephritidae) in China and Southeast Asia. J. Econ. Entomol..

[9] Dias N., Zotti M., Montoya P., Carvalho I. (2018). Fruit fly management research: A systematic review of monitoring and control tactics in the world. Crop Prot..

[10] Garcia F., Ovruski S.M., Suárez L., Cancino J., Liburd O.E. (2020). Biological control of Tephritid fruit flies in the Americas and Hawaii: A review of the use of parasitoids and predators. Insects.

[11] Ao G., Lin J., Liu X., Ji Q. (2019). Advances in research on insecticide resistance in the Tephritidae. Chin. J. Appl. Entomol..

[12] Baronio C.A., Bernardi D., Schutze I.X., Baldin M.M., Botton M. (2019). Toxicities of insecticidal toxic baits to control *Ceratitis capitata* (Diptera: Tephritidae): Implications for field management. J. Econ. Entomol..

[13] Jaffar S., Rizvi S.A.H., Lu Y. (2023). Understanding the invasion, ecological adaptations, and management strategies of *Bactrocera dorsalis* in China: A Review. Horticulturae.

[14] Pérez-Staples D., Díaz-Fleischer F., Montoya P. (2021). The Sterile Insect Technique: Success and Perspectives in the Neotropics. Neotrop. Entomol..

[15] Liu H., Zhang D., Xu Y., Wang L., Cheng D., Qi Y., Zeng L., Lu Y. (2018). Invasion, expansion, and control of *Bactrocera dorsalis* (Hendel) in China. J. Integr. Agric..

[16] Diouf E.G., Brévault T., Ndiaye S., Faye E., Chailleux A., Diatta P., Piou C. (2022). An agent-based model to simulate the boosted Sterile Insect Technique for fruit fly management. Ecol. Model..

[17] Cladera J.L., Vilardi J.C., Juri M., Paulin L.E., Lanzavecchia S.B. (2014). Genetics and biology of *Anastrepha fraterculus*: Research supporting the use of the sterile insect technique (SIT) to control this pest in Argentina. BMC Genet..

[18] Mau R.F.L., Jang E.B., Vargas R. (2007). The Hawaii area-wide fruit fly pest management programme: Influence of partnerships and a good education programme. Area-Wide Control Insect Pests.

[19] Haq I.U., Abd-Alla A., Tomas U.S., Meza J.S., Bourtzis K., Cáceres C. (2019). Cryopreservation of the Mediterranean fruit fly (Diptera: Tephritidae) VIENNA 8 genetic sexing strain: No effect on large scale production of high quality sterile males for SIT applications. PLoS ONE.

[20] Li S., Zhang K., Wen J., Zeng Y., Deng Y., Hu Q., Weng Q. (2023). Molecular mechanism of male sterility induced by 60Co γ-Rays on *Plutella xylostella* (Linnaeus). Molecules.

[21] Fuller M.T. (1998). Genetic control of cell proliferation and differentiation in *Drosophila* spermatogenesis. Semin. Cell Dev. Biol..

[22] Neto F.T., Bach P.V., Najari B.B., Li P.S., Goldstein M. (2016). Spermatogenesis in humans and its affecting factors. Semin. Cell Dev. Biol..

[23] Michiels F., Gasch A., Kaltschmidt B., Renkawitz-Pohl R. (1989). A 14 bp promoter element directs the testis specificity of the *Drosophila beta 2 tubulin* gene. EMBO J..

[24] Jan S.Z., Hamer G., Repping S., de Rooij D.G., van Pelt A.M., Vormer T.L. (2012). Molecular control of rodent spermatogenesis. Biochim. Biophys. Acta.

[25] Klowden M.J., Resh V.H., Cardé R.T. (2009). Chapter 189—Oviposition Behavior. Encyclopedia of Insects.

[26] Sohail A., Al-Dalali S., Wang J., Xie J., Shakoor A., Sailimuhan A., Shah H., Patil P. (2022). Aroma compounds identified in cooked meat: A review. Food Res. Int..

[27] Yang J.H., Hayano M., Griffin P.T., Amorim J.A., Bonkowski M.S., Apostolides J.K., Salfati E.L., Blanchette M., Munding E.M., Bhakta M. (2023). Loss of epigenetic information as a cause of mammalian aging. Cell.

[28] Kemphues K.J., Raff R.A., Kaufman T.C., Raff E.C. (1979). Mutation in a structural gene for a *beta-tubulin* specific to testis in *Drosophila melanogaster*. Proc. Natl. Acad. Sci. USA.

[29] Smith R.C., Walter M.F., Hice R.H., O’Brochta D.A., Atkinson P.W. (2007). Testis-specific expression of the *beta2 tubulin* promoter of *Aedes aegypti* and its application as a genetic sex-separation marker. Insect Mol. Biol..

[30] Michiels F., Wolk A., Renkawitz-Pohl R. (1991). Further sequence requirements for male germ cell-specific expression under the control of the 14 bp promoter element (*beta 2UE1*) of the *Drosophila beta 2 tubulin* gene. Nucleic Acids Res..

[31] Santel A., Kaufmann J., Hyland R., Renkawitz-Pohl R. (2000). The initiator element of the *Drosophila beta2 tubulin* gene core promoter contributes to gene expression in vivo but is not required for male germ-cell specific expression. Nucleic Acids Res..

[32] Khan S.A., Jakes E., Myles K.M., Adelman Z.N. (2021). The *β(2)Tubulin*, Rad50-ATPase and enolase cis-regulatory regions mediate male germline expression in *Tribolium castaneum*. Sci. Rep..

[33] Scolari F., Schetelig M.F., Bertin S., Malacrida A.R., Gasperi G., Wimmer E.A. (2008). Fluorescent sperm marking to improve the fight against the pest insect *Ceratitis capitata* (Wiedemann; Diptera: Tephritidae). New Biotechnol..

[34] Thaler C.D., Carstens K., Martinez G., Stephens K., Cardullo R.A. (2023). Using the *Culex pipiens* sperm proteome to identify elements essential for mosquito reproduction. PLoS ONE.

[35] Mita K., Nenoi M., Morimyo M., Tsuji H., Ichimura S., Sawai M., Hamana K. (1995). Expression of the *Bombyx mori beta-tubulin*-encoding gene in testis. Gene.

[36] Li A.M., He W.Z., Wei J.L., Chen Z.L., Liao F., Qin C.X., Pan Y.Q., Shang X.K., Lakshmanan P., Wang M. (2022). Transcriptome profiling reveals genes related to sex determination and differentiation in Sugarcane Borer (*Chilo sacchariphagus* Bojer). Insects.

[37] Liao J., Chen J., Liu D., Li J., Chen J., Sun C., Wei H., Asad M., Yang G. (2025). Molecular and functional characterization of a *β-tubulin* gene in *Plutella xylostella*. Int. J. Biol. Macromol..

[38] Durant A.C., Donini A. (2020). Ammonium transporter expression in sperm of the disease vector *Aedes aegypti* mosquito influences male fertility. Pro. Natl. Acad. Sci. USA.

[39] Sun X., Wang X., Shi K., Lyu X., Sun J., Raikhel A.S., Zou Z. (2024). Leucine aminopeptidase1 controls egg deposition and hatchability in male *Aedes aegypti* mosquitoes. Nat. Commun..

[40] Wu S., Ran L., Zhang T., Li Y., Xu Y., Li Y., Liu H., Wang J. (2024). *BdTTLL3B*-mediated polyglycylation is involved in the spermatogenesis in *Bactrocera dorsalis*. Int. J. Biol. Macromol..

[41] Bielke W., Blaschke R.J., Miescher G.C., Zürcher G., Andres A.C., Ziemiecki A. (1994). Characterization of a novel murine testis-specific serine/threonine kinase. Gene.

[42] Kueng P., Nikolova Z., Djonov V., Hemphill A., Rohrbach V., Boehlen D., Zuercher G., Andres A.C., Ziemiecki A. (1997). A novel family of serine/threonine kinases participating in spermiogenesis. J. Cell Biol..

[43] Zuercher G., Rohrbach V., Andres A.C., Ziemiecki A. (2000). A novel member of the testis specific serine kinase family, tssk-3, expressed in the Leydig cells of sexually mature mice. Mech. Dev..

[44] Hao Z., Jha K.N., Kim Y.H., Vemuganti S., Westbrook V.A., Chertihin O., Markgraf K., Flickinger C.J., Coppola M., Herr J.C. (2004). Expression analysis of the human testis-specific serine/threonine kinase (TSSK) homologues. A TSSK member is present in the equatorial segment of human sperm. Mol. Hum. Reprod..

[45] Xu B., Hao Z., Jha K.N., Digilio L., Urekar C., Kim Y.H., Pulido S., Flickinger C.J., Herr J.C. (2007). Validation of a testis specific serine/threonine kinase [TSSK] family and the substrate of TSSK1 & 2, TSKS, as contraceptive targets. Soc. Reprod. Fertil. Suppl..

[46] Xu B., Hao Z., Jha K.N., Zhang Z., Urekar C., Digilio L., Herr J.C. (2008). Targeted deletion of Tssk1 and 2 causes male infertility due to haploinsufficiency. Dev. Biol..

[47] Peng J., Sun A., Zheng J., Zhang N., Zhang X., Gao G. (2025). Testis-specific serine/threonine kinase dTSSK2 regulates sperm motility and male fertility in *Drosophila*. Commun. Biol..

[48] Cruz C., Tayler A., Whyard S. (2018). RNA interference-mediated knockdown of male fertility genes in the Queensland fruit fly *Bactrocera tryoni* (Diptera: Tephritidae). Insects.

[49] Sohail S., Tariq K., Zheng W., Ali M.W., Peng W., Raza M.F., Zhang H. (2019). RNAi-mediated knockdown of *Tssk1* and *Tektin1* genes impair male fertility in *Bactrocera dorsalis*. Insects.

[50] Zhai X.D., Zhang S.Y., Chen D., Li W.J., Wang J.J., Wei D. (2023). Comparative multi-tissue analyses identify testis-specific serine/threonine protein kinase (TSSK) genes involved in male fertility in the melon fly *Zeugodacus cucurbitae*. Pest Manag. Sci..

[51] Wei Z., Wang Y., Zheng K., Wang Z., Liu R., Wang P., Li Y., Gao P., Akbari O.S., Yang X. (2024). Loss-of-function in testis-specific serine/threonine protein kinase triggers male infertility in an invasive moth. Commun. Biol..

[52] Liu P., Li Z., Zhang Q., Qiao J., Zheng C., Zheng W., Zhang H. (2024). Identification of testis development-related genes by combining Iso-Seq and RNA-Seq in *Zeugodacus tau*. Front. Cell Dev. Biol..

[53] Faber N.R., Ashok K., Venkatesan T., Wertheim B., Bulgarella M. (2026). Leveraging advances in RNAi and CRISPR for improved biological pest control. Curr. Opin. Insect Sci..

[54] Li Z., Liu Y., Liang Y., Pan T., Liu J. (2026). RNA interference-based pesticides: Mechanism, application, and commercialization in sustainable pest management. Pestic. Biochem. Physiol..

[55] Baum J.A., Bogaert T., Clinton W., Heck G.R., Feldmann P., Ilagan O., Johnson S., Plaetinck G., Munyikwa T., Pleau M. (2007). Control of coleopteran insect pests through RNA interference. Nat. Biotechnol..

[56] Zhu F., Xu J., Palli R., Ferguson J., Palli S.R. (2011). Ingested RNA interference for managing the populations of the Colorado potato beetle, *Leptinotarsa decemlineata*. Pest Manag. Sci..

[57] Spit J., Philips A., Wynant N., Santos D., Plaetinck G., Vanden Broeck J. (2017). Knockdown of nuclease activity in the gut enhances RNAi efficiency in the Colorado potato beetle, *Leptinotarsa decemlineata*, but not in the desert locust, *Schistocerca gregaria*. Insect Biochem. Mol. Biol..

[58] Zhu K.Y., Palli S.R. (2020). Mechanisms, applications, and challenges of insect RNA interference. Annu. Rev. Entomol..

[59] Taning C.N.T., Mezzetti B., Kleter G., Smagghe G., Baraldi E. (2021). Does RNAi-based technology fit within EU sustainability goals?. Trends Biotechnol..

[60] Narva K., Toprak U., Alyokhin A., Groves R., Jurat-Fuentes J.L., Moar W., Nauen R., Whipple S., Head G. (2025). Insecticide resistance management scenarios differ for RNA-based sprays and traits. Insect Mol. Biol..

[61] Airs P.M., Bartholomay L.C. (2017). RNA interference for mosquito and mosquito-borne disease control. Insects.

[62] Liu J., He Q., Lin X., Smagghe G. (2025). Recent progress in nanoparticle-mediated RNA interference in insects: Unveiling new frontiers in pest control. J. Insect Physiol..

[63] Li X., Zhang M., Zhang H. (2011). RNA interference of four genes in adult *Bactrocera dorsalis* by feeding their dsRNAs. PLoS ONE.

[64] Dong Y.C., Wang Z.J., Chen Z.Z., Clarke A.R., Niu C.Y. (2016). *Bactrocera dorsalis* male sterilization by targeted RNA interference of spermatogenesis: Empowering sterile insect technique programs. Sci. Rep..

[65] Volpe G., Mazzucchiello S.M., Rosati N., Lucibelli F., Varone M., Baccaro D., Mattei I., Di Lelio I., Becchimanzi A., Giordano E. (2024). Simultaneous silencing of gut nucleases and a vital target gene by adult dsRNA feeding enhances RNAi efficiency and mortality in *Ceratitis capitata*. Insects.

[66] Ortolá B., Urbaneja A., Eiras M., Pérez-Hedo M., Daròs J.A. (2024). RNAi-mediated silencing of Mediterranean fruit fly (*Ceratitis capitata*) endogenous genes using orally-supplied double-stranded RNAs produced in *Escherichia coli*. Pest Manag. Sci..

[67] Dias N., Cagliari D., Kremer F.S., Rickes L.N., Nava D.E., Smagghe G., Zotti M. (2019). The south American fruit fly: An important pest insect with RNAi-sensitive larval stages. Front. Physiol..

[68] Tayler A., Heschuk D., Giesbrecht D., Park J.Y., Whyard S. (2019). Efficiency of RNA interference is improved by knockdown of dsRNA nucleases in Tephritid fruit flies. Open Biol..

[69] Ahmad S., Jamil M., Jaworski C.C., Luo Y. (2024). Double-stranded RNA degrading nuclease affects RNAi efficiency in the Melon Fly, *Zeugodacus cucurbitae*. J. Pest Sci..

[70] Gregoriou M.E., Mathiopoulos K.D. (2020). Knocking down the sex peptide receptor by dsRNA feeding results in reduced oviposition rate in olive fruit flies. Arch. Insect Biochem. Physiol..

[71] Ali M.W., Zheng W., Sohail S., Li Q., Zheng W., Zhang H. (2017). A genetically enhanced sterile insect technique against the fruit fly, *Bactrocera dorsalis* (Hendel) by feeding adult double-stranded RNAs. Sci. Rep..

[72] Al Baki M.A., Vatanparast M., Kim Y. (2020). Male-biased adult production of the striped fruit fly, *Zeugodacus scutellata*, by feeding dsRNA specific to *Transformer-2*. Insects.

[73] Volpe G., Mazzucchiello S.M., De Falco D., Torrente D., Liguori S., Rosati N., Baccaro D., Mazzeo M., Bertolotto F., Sangle H. (2026). DsiRNA-mediated silencing of *Ceratitis capitata transformer* or *transformer-2* leads to masculinization of XX embryos and systemic gene silencing in ovaries. Insect Sci..

[74] Mysore K., Sun L., Tomchaney M., Sullivan G., Adams H., Piscoya A.S., Severson D.W., Syed Z. (2015). Duman-Scheel M: siRNA-mediated silencing of *doublesex* during female development of the dengue vector mosquito *Aedes aegypti*. PLoS Negl. Trop. Dis..

[75] Taracena M.L., Hunt C.M., Benedict M.Q., Pennington P.M., Dotson E.M. (2019). Downregulation of female *doublesex* expression by oral-mediated RNA interference reduces number and fitness of *Anopheles gambiae* adult females. Parasites Vectors.

[76] Zhang X., Peng J., Wu M., Sun A., Wu X., Zheng J., Shi W., Gao G. (2023). Broad phosphorylation mediated by testis-specific serine/threonine kinases contributes to spermiogenesis and male fertility. Nat. Commun..

[77] Livak K., Schmittgen T.D. (2001). Analysis of Relative Gene Expression Data Using Real-Time Quantitative PCR and the 2^−ΔΔC_T_^ Method. Methods.

[78] Liu L., Lu X., Fan Z., Deng J., Zhang S., Zhang L., Zha X. (2024). TPCA-1 compound, inhibiting testis-specific serine/threonine protein kinase 3 for potential male sterile in *Bombyx mori*. Pest Manag. Sci..

[79] Peng L., Bian H.M., Zheng J.H., Tun L.W., Zou M.M., Cao M.H., Cui J.D., Vasseur L., Qian Y.R., Huang M.Q. (2026). Impacts of testis-specific serine/threonine protein kinase 3 on eupyrene spermatogenesis and contributes to male sterility in *Plutella xylostella*. Pest Manag. Sci..

[80] Meghwanshi K.K., Choudhary C., Rohilla P., Dixit R., Saxena V., Shukla J.N. (2025). Testis-specific serine/threonine kinase 3 regulates the size of sperm reservoir in *Anopheles stephensi*. Mol. Genet. Genom..

[81] Hanks S.K., Quinn A.M., Hunter T. (1988). The protein kinase family: Conserved features and deduced phylogeny of the catalytic domains. Science.

[82] Li W.J., Wei D., Han H.L., Song Y.J., Wang Y., Xu H.Q., Smagghe G., Wang J.J. (2021). lnc94638 is a testis-specific long non-coding RNA involved in spermatozoa formation in *Zeugodacus cucurbitae* (Coquillett). Insect Mol. Biol..

[83] Harris A.F., Nimmo D., Mckemey A.R., Kelly N., Scaife S., Donnelly C.A., Beech C., Petrie W.D., Alphey L. (2011). Field performance of engineered male mosquitoes. Nat. Biotechnol..

