# Supplementary material for: RNA Interference Targeting Testis-Specific Serine/Threonine Protein Kinase 1 (TSSK1) Gene Triggers Male Infertility in Zeugodacus tau
Source: Insects. 2026 May 12;17(5):492. doi: 10.3390/insects17050492 (PMC13206916; doi:10.3390/insects17050492)
Supplement: Supplementary file 1 [file insects-17-00492-s001.zip › Table S1.pdf]

Table S1 Primers used in our study

Primer names	Sequence 5’ to 3’
qPCR primers:	
ZtTSSK1-F	CGCAATAAGAAGTTCGCATT
ZtTSSK1-R	TTTCACGTAAATTCCAACGC
rpl32-F	TTAGGTCTATTCGTTCTCCTGTG
rpl32-R	TTGGTCCACAATGTCCGTGA
dsRNA synthesis primers:	
ds*ZtTSSK1*-F	GGATCCTAATACGACTCACTATAGGCTCCAATCGCCAACTGAA
ds*ZtTSSK1*-R	GGATCCTAATACGACTCACTATAGGTGATCGTCCAGCAACTTG
ds*GFP*-F	GGATCCTAATACGACTCACTATAGGGCAACGGTGTGGACTTTGAC
ds*GFP*-R	GGATCCTAATACGACTCACTATAGGGCGGCTGGTTCTTCAGATAGTT
