# Supplementary figures and images for: RNA Interference Targeting Testis-Specific Serine/Threonine Protein Kinase 1 (TSSK1) Gene Triggers Male Infertility in Zeugodacus tau
Source: Insects. 2026 May 12;17(5):492. doi: 10.3390/insects17050492 (PMC13206916; doi:10.3390/insects17050492)

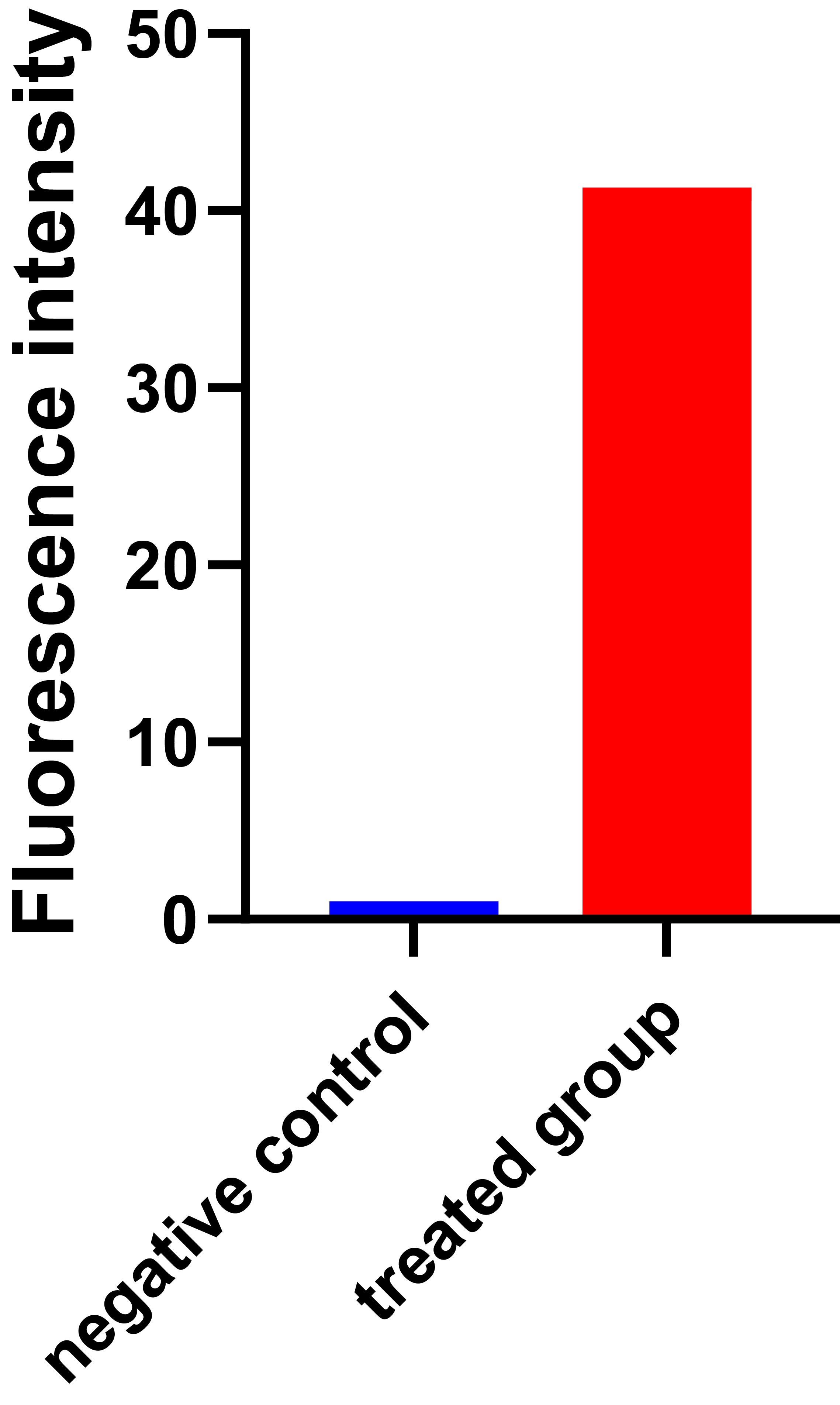

Supplement: Supplementary file 1 [file insects-17-00492-s001.zip › Figure S1.jpg]

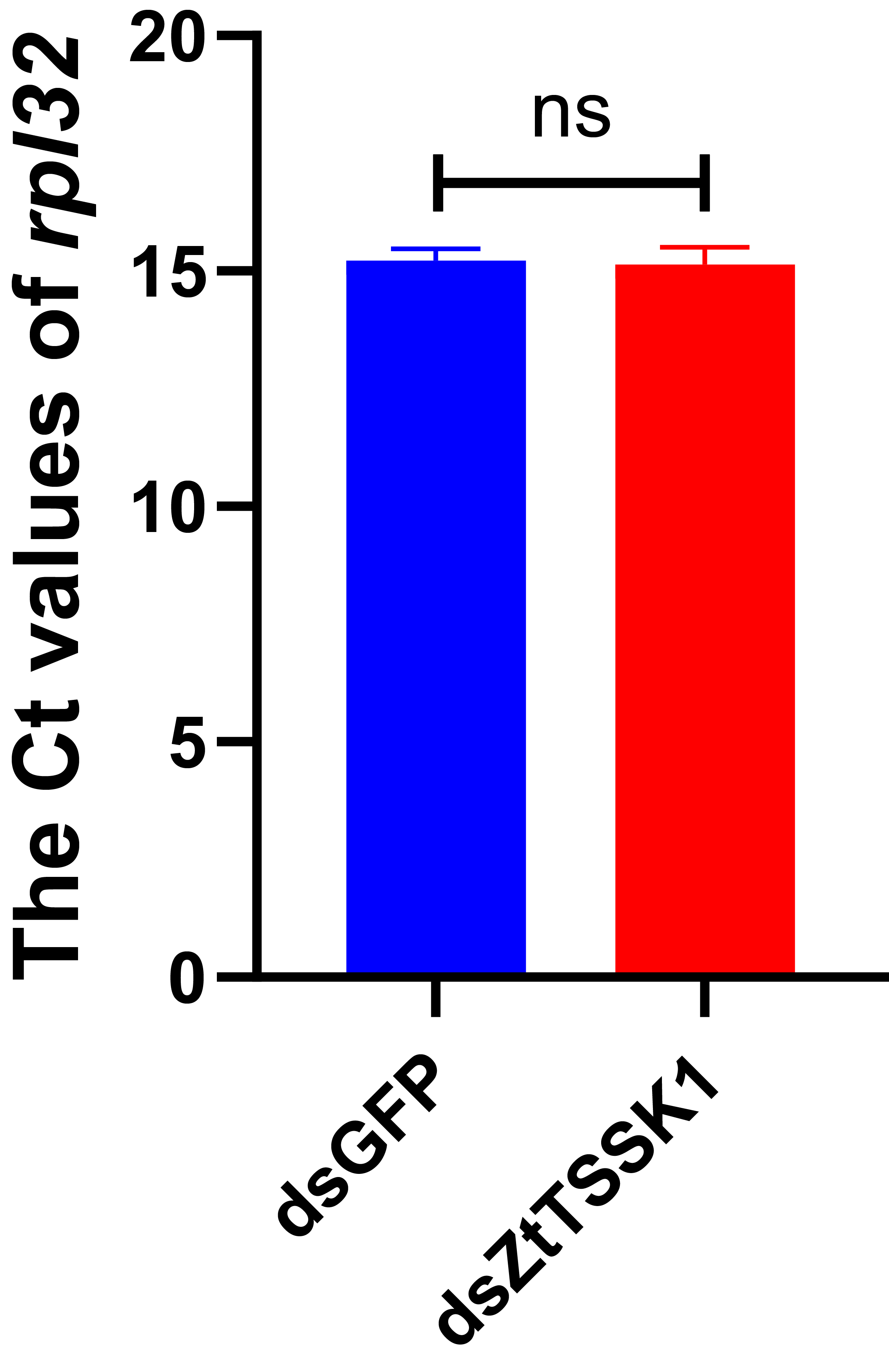

Supplement: Supplementary file 1 [file insects-17-00492-s001.zip › Figure S2.jpg]

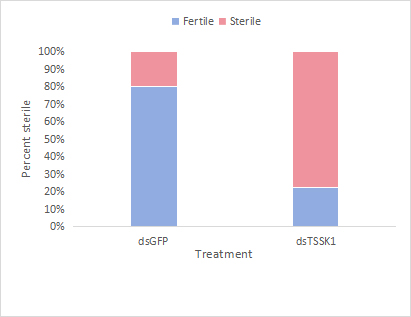

Supplement: Supplementary file 1 [file insects-17-00492-s001.zip › Figure S3.jpg]
